# Effects of Rapid Weight Loss on the Immune System in Combat Sports Athletes: A Systematic Review

**DOI:** 10.3390/ijms27010508

**Published:** 2026-01-03

**Authors:** Hae Sung Lee

**Affiliations:** Department of Physical Education, College of Education, Wonkwang University, Iksan-si 54538, Republic of Korea; haesung2@wku.ac.kr; Tel.: +82-63-850-6953

**Keywords:** rapid weight loss, combat sports athletes, immune system

## Abstract

Rapid weight loss (RWL) is a common strategy among combat sports athletes aiming for a competitive advantage. However, it imposes significant immunological stress that compromises both innate and adaptive immune defenses. This systematic review synthesizes current experimental and mechanistic evidence on the effects of RWL in combat sports, focusing on cellular immunity, neuroendocrine regulation, and inflammatory pathways. Acute RWL activates the hypothalamic–pituitary–adrenal axis, elevating plasma cortisol and suppressing lymphocyte proliferation, T-cell function, and natural killer cell cytotoxicity. Although neutrophil counts increase, their phagocytic and oxidative burst capacities decline, reflecting impaired host defense. Monocyte and macrophage systems shift toward proinflammatory phenotypes, while mucosal immunity is weakened by reductions in secretory immunoglobulin A, leading to increased upper respiratory tract infection risk. The magnitude and speed of weight loss are critical determinants of immune dysfunction, with reductions exceeding 5% of body mass producing particularly severe consequences. Evidence-based intervention strategies—including gradual weight management, nutritional optimization, and biomarker monitoring—are essential to mitigate immunosuppression and safeguard athlete health. This review highlights key gaps in combat sports-specific protocols and proposes integrated approaches to preserve immune competence and optimize performance.

## 1. Introduction

The rapid weight loss (RWL) is a widespread strategy among combat sports athletes aiming to compete in lower weight categories and gain a competitive advantage [1]. This practice typically involves reducing body weight by 3–6% within 3–5 days before competition through severe caloric restriction, dehydration, and excessive exercise [2]. Although such methods facilitate quick weight reductions, they impose multifactorial immunological stress, compromising both innate and adaptive defense mechanisms [3].

The hypothalamic–pituitary–adrenal (HPA) axis activation induced by acute energy deficits elevates cortisol concentrations, which suppresses lymphocyte proliferation, impairs T-helper cell function, and dysregulates cytokine production, notably reducing interleukin (IL)-2 synthesis critical for T-cell activation [4]. Concurrently, severe dehydration decreases plasma volume, concentrates circulating neutrophils, and paradoxically lowers their phagocytic capacity through impaired oxidative burst, thereby increasing infection susceptibility during the “open-window” of post-exercise immunosuppression [5]. Secretory immunoglobulin A (sIgA) levels decrease after RWL, which has been associated with increased incidence of upper respiratory tract infections (URTI) in elite taekwondo athletes during intense training and competition periods [5]. Moreover, natural killer (NK) cell cytotoxicity also tends to decrease following body mass reductions exceeding approximately 5%, although the exact threshold varies among studies, and recovery time may extend up to 24 h post-competition [6]. In addition to this impairment of cell-mediated immunity, RWL induces a systemic proinflammatory state, elevating tumor necrosis factor (TNF)-α and IL-6 levels; when repeated, these elevations may precipitate chronic immune dysregulation and oxidative stress [4]. Lipid peroxidation markers, including malondialdehyde, increase following RWL, indicating elevated oxidative stress. Meanwhile, activities of antioxidant enzymes such as superoxide dismutase may fluctuate, suggesting impaired redox homeostasis in these athletes [7]. Despite extensive investigations into RWL effects in general athletic populations, a focused synthesis addressing the unique immune–metabolic challenges of combat sports protocols remains lacking. In particular, the timing and magnitude of cytokine responses, complement activation, and mucosal antibody dynamics under severe weight-cutting regimens have not been integrated for these athletes. Given the combat sports reliance on lower-body explosiveness and rapid recovery between bouts, understanding the nuanced interplay between body weight-cutting methods and immune function is essential. Defining the “open window” of post-cut immunosuppression—characterized by transient lymphopenia, neutrophil dysfunction, reduced NK cell cytotoxicity, and impaired sIgA—is essential to understanding increased infection risk in weight-categorical competition. Moreover, the interplay between hypothalamic–pituitary–adrenal axis activation and redox imbalance warrant targeted examination. So far, while numerous reviews have characterized RWL in combat sports, few have integrally dissected the unique immunometabolism stressors inherent to combat sports-specific RWL protocols. This review integrates mechanistic insights from HPA axis activation, cytokine kinetics, and redox homeostasis—areas underrepresented in prior literature—to provide a targeted synthesis of innate and adaptive immune perturbations specific to combat sports athletes, thereby filling a critical gap in sports immunology research.

Therefore, this review aims to critically examine current knowledge on the immunological consequences of RWL in combat sports athletes, with a particular focus on alterations in innate and adaptive defense mechanisms, endocrine and molecular regulatory pathways, and the proinflammatory milieu induced by acute body weight reduction. By identifying key limitations and gaps in existing research—such as the lack of combat sports-specific protocols and inconsistent cytokine kinetics—we propose informed hypotheses and novel perspectives on underexplored areas, including mucosal immunity and complement system dynamics. Ultimately, this review seeks to deepen our understanding of how rapid weight-cutting practices compromise immune competence in weight-categorical sports and to provide evidence-based, athlete-centered weight-management strategies that safeguard immunological health while optimizing competitive performance.

## 2. Methods

This systematic review was conducted in strict accordance with the Preferred Reporting Items for Systematic Reviews and Meta-Analyses (PRISMA) guidelines to enhance the rigor, transparency, and reproducibility of the research process (Supplementary Table S1) [8]. The review protocol was not registered in a prospective register such as PROSPERO. The review aimed to comprehensively synthesize existing evidence on the immunological effects of RWL in combat sports athletes, while integrating mechanistic insights from related biomedical fields due to the limited availability of direct experimental studies on the target population.

### 2.1. Search Strategy and Selection Criteria

A comprehensive and integrative literature search was carried out across four major electronic databases: PubMed, Scopus, Web of Science, and Google Scholar. The search covered all articles published from January 2000 through September 2025 to encompass research reflecting weight loss. Search terms were developed based on a preliminary scoping review and consultation with domain experts to ensure relevance and completeness. The search query combined Medical Subject Headings (MeSH) and free-text keywords, using Boolean operators (AND, OR) for optimal balance between sensitivity and specificity. Key search terms included: “rapid weight loss”, “weight loss”, “combat sports”, “taekwondo”, “judo”, “wrestling”, “immune response”, “cytokines”, “natural killer cells”, “sIgA”, and “inflammation” (Table 1). Additionally, reference lists of identified articles and relevant reviews were also screened to locate additional pertinent studies.

### 2.2. Eligibility Criteria for Study Selection

Study eligibility was defined using the PICOS framework as follows:

- Population: Active combat sports athletes (e.g., taekwondo, judo, wrestling, boxing).
- Intervention: Exposure to RWL protocols, customarily defined as ≥3% body mass reduction over a 7-day or shorter period.
- Comparator: Pre-intervention baseline measurements, control groups, or longitudinal repeated measures.
- Outcomes: Immune function parameters measured directly (e.g., immune cell counts, cytokine profiles, mucosal immunity markers) or inferred from related biomolecular indicators.
- Study Design: Peer-reviewed randomized controlled trials, quasi-experimental studies, pre-post intervention designs, and observational cohorts.
- Language: English.

Exclusion criteria comprised studies involving long-term weight reduction (>4 weeks), non-athlete populations, absence of immune-related outcomes, or grey literature lacking peer review.

### 2.3. Multi-Level Reviews and Cross-Checks

Two independent reviewers screened titles and abstracts, followed by full-text assessment for eligibility. Discrepancies in selection were resolved via consensus or consultation with a third reviewer. Data extraction employed a structured form capturing study identifiers, participant demographics, weight loss details (magnitude, duration, methods), immune parameters assessed, and principal findings.

Because direct experimental evidence on immune changes following RWL in combat sports athletes is sparse, this review supplemented mechanistic interpretation by integrating data from related fields such as obesity, oncology, general adult health, aging populations, pediatric investigations, and animal experimental models. Mechanisms underlying observed immune alterations were thus explored through this broader biomedical literature, enabling a comprehensive understanding of potential physiological pathways relevant to the athletic context.

### 2.4. Quality Assessment and Risk of Bias

**Figure 1:**
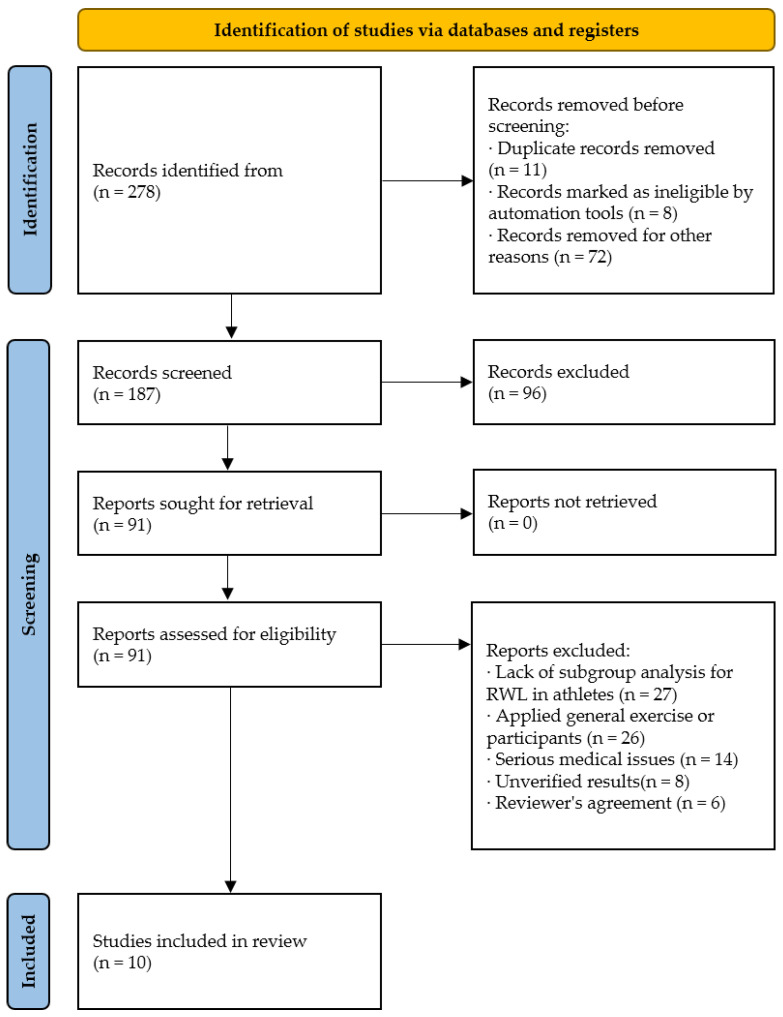
Study selection and screening protocol following PRISMA guidelines.

The methodological quality of the 10 included studies was rigorously assessed using domain-specific instruments. Specifically, for Randomized Controlled Trials (e.g., [9,10,11,12]), the Cochrane Risk of Bias 2 tool was employed, and for Non-Randomized Studies (e.g., [4,13,14,15]), the Newcastle–Ottawa Scale was utilized. Two evaluators independently rated risk of bias and reporting quality, and divergent assessments were systematically reconciled by consensus or adjudication (Figure 1).

## 3. Results

**Figure 2:**
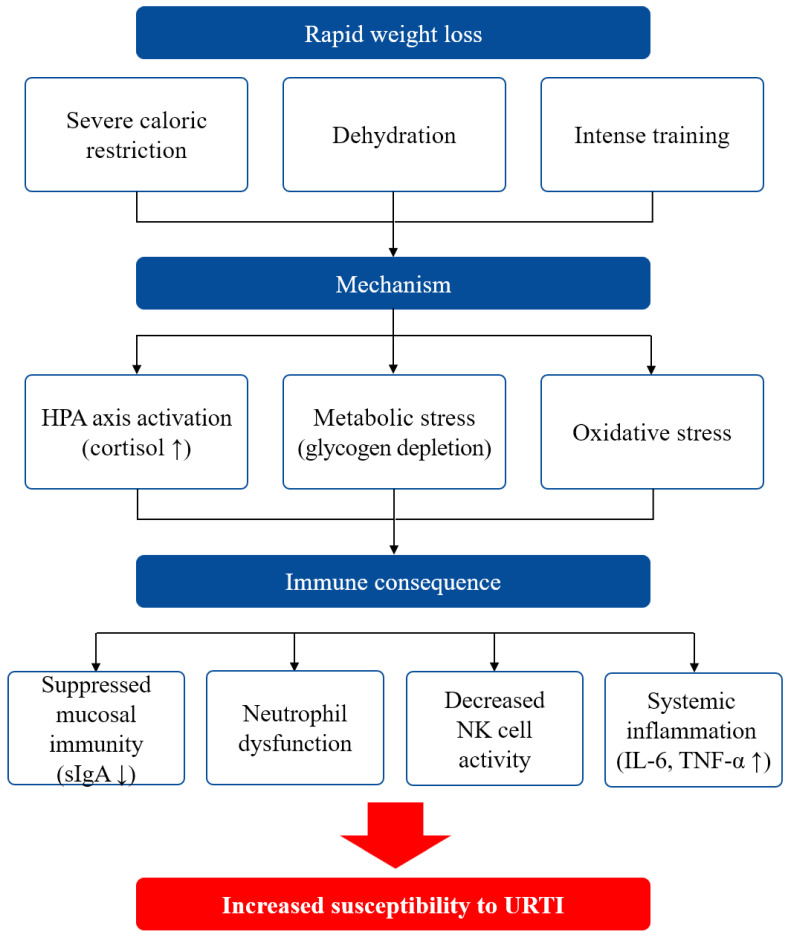
Conceptual model of immune dysfunction induced by rapid weight loss in combat sports athletes. HPA: hypothalamic–pituitary–adrenal axis; sIgA: secretory immunoglobulin A; NK: natural killer cells; TNF-α: tumor necrosis factor-alpha; IL-6: interleukin-6; URTI: upper respiratory tract infections.

RWL is a widespread practice among combat sport athletes who seek to compete in lower weight categories by reducing 5–10% of their body weight within days to weeks before competition. While this strategy may confer competitive advantages, accumulating experimental evidence demonstrates that such aggressive weight reduction protocols induce substantial immunological perturbations that elevate susceptibility to opportunistic infections (Figure 2).

### Changes in the Immune System Due to Rapid Weight Loss

Hiraoka et al. [12] investigated the immunological status of 30 university judo athletes during a pre-competition weight loss period, dividing participants into three groups based on the magnitude of weight reduction: control (no weight loss), under 5% weight loss, and over 5% weight loss groups. The researchers observed that athletes losing more than 5% of body weight exhibited significantly decreased sIgA secretion rates at 3 days and 1 day before competition compared to the control group. Notably, saliva flow rate declined in parallel with sIgA secretion velocity, decreasing from baseline values 2 weeks prior to competition. The authors attributed these changes to dehydration-induced alterations in mucosal immunity, wherein reduced saliva volume paradoxically increased sIgA concentration while decreasing the absolute secretion rate—a critical determinant of mucosal barrier function. This immunological compromise manifested clinically, as the over 5% weight loss group reported markedly elevated incidence of URTI symptoms, including 6 cases of lassitude, 5 cases of runny nose, 3 cases of sore throat, and 1 headache, whereas the control group reported no such symptoms. The investigators concluded that weight reduction exceeding 5% compromises oral immunity through dehydration-mediated suppression of sIgA secretion, thereby increasing vulnerability to respiratory infections.

Complementing these findings on mucosal immunity, Abedelmalek et al. [4] examined systemic inflammatory and hormonal responses during RWL in 11 male judokas who underwent 7 days of caloric restriction (CR), reducing energy intake from 3475 ± 247.6 to 2192 ± 235.6 kcal/day—approximately a 37% reduction. This intervention resulted in a 4.2 ± 0.5% decrease in body weight (from 75.9 ± 3.1 to 72.73 ± 3.1 kg) accompanied by substantial body water loss (from 47.72 ± 1.9 to 41.8 ± 1.8 kg). The researchers measured plasma concentrations of proinflammatory cytokines before, immediately after, and 60 min following the Special Judo Fitness Test under both baseline and caloric restriction conditions. IL-6 demonstrated a pronounced response to both exercise and energy restriction, with significantly elevated levels during CR compared to baseline. Similarly, TNF-α concentrations increased immediately post-exercise and remained significantly higher during CR relative to baseline. The authors proposed that glycogen depletion consequent to severe energy restriction serves as a primary stimulus for IL-6 release from skeletal muscle, while the elevation of proinflammatory cytokines collectively reflects immune system degradation and heightened inflammatory stress. Mechanistically, this secretion of IL-6 from skeletal muscle is tightly regulated by intramuscular glycogen availability. Glycogen depletion acts as a potent metabolic stressor that activates energy-sensing signaling pathways, specifically AMP-activated protein kinase and p38 mitogen-activated protein kinase [16]. Upon activation, these kinases translocate to the nucleus and stimulate transcriptional factors to upregulate IL-6 gene expression [16]. The released IL-6 then functions in a hormone-like manner (“myokine”) to enhance hepatic glucose production and lipolysis, thereby attempting to maintain substrate homeostasis during states of severe energy deficit [16]. Consequently, the elevated IL-6 observed in RWL is not solely an inflammatory marker but also reflects a compensatory metabolic response to glycogen insufficiency [4,16]. This study by Abedelmalek et al. further revealed profound hormonal dysregulation accompanying RWL. Cortisol concentrations increased significantly during CR at all measurement time points compared to baseline, whereas testosterone levels decreased, and growth hormone increased. The investigators attributed the elevated cortisol to the physiological stress imposed by restrained eating and low carbohydrate intake, noting that hypercortisolemia exerts potent immunosuppressive effects that increase susceptibility to infections [17,18]. Concurrently, the reduction in testosterone—a hormone with recognized immune-supportive properties—further compromised immune function when combined with dehydration and intensive exercise demands [19]. Although testosterone is often characterized as immunosuppressive in high doses, physiological concentrations exert critical immunomodulatory effects that are essential for homeostasis. Testosterone acts through androgen receptors expressed on leukocytes, including macrophages and neutrophils, to inhibit the nuclear translocation of NF-κB, thereby suppressing the excessive production of pro-inflammatory cytokines such as TNF-α and IL-6 [20]. Furthermore, it shifts the T-helper cell balance away from a pro-inflammatory Th1 profile and promotes the expression of anti-inflammatory mediators like IL-10 [4]. Therefore, the hypotestosteronemia induced by RWL removes this protective “brake” on inflammation, exacerbating the systemic pro-inflammatory state and delaying tissue recovery [20]. Cellular immune parameters mirrored these hormonal perturbations, with leukocyte counts declining significantly during CR relative to baseline, while neutrophils increased post-exercise in both conditions. The authors concluded that 7-day weight loss programs degrade both immune and endocrine functions through the synergistic effects of energy restriction, dehydration, and intensified training, thereby predisposing athletes to infectious diseases such as URTI [4,13].

Investigating whether plasma glutamine depletion mediates weight-loss-induced immunosuppression, Tritto et al. [10] conducted a double-blind, randomized, placebo-controlled study in 23 male combat athletes who reduced body weight by approximately 8% over 10 days preceding competition. The control group maintained stable body weight (−0.6 ± 1.4%). The majority of weight-reducing athletes—67% in the glutamine group and 72% in the placebo group—reported URTI symptoms, contrasting sharply with the 25% incidence in controls. Analysis of immune cell function revealed an increase in neutrophil phagocytic activity following weight loss, with fluorescence intensity values rising from 5251 ± 2986 at baseline to 17,428 ± 22,374 at pre-competition and 21,125 ± 21,934 at post-competition in the placebo group, and from 6096 ± 3549 to 11,029 ± 17,113 and 28,186 ± 21,032 in the glutamine group. Monocyte phagocytic activity exhibited minor fluctuations, while oxidative burst capacity in both cell types remained unchanged. Critically, plasma glutamine concentrations remained stable throughout the study period, with no athlete exhibiting levels below the 400 μmol/L threshold associated with immune dysfunction (placebo baseline: 791 ± 229 μmol/L; glutamine baseline: 830 ± 258 μmol/L). The researchers concluded that RWL induced immunosuppression occurs independently of plasma glutamine depletion, proposing instead that intensified training loads—which reduce circulating and mucosal immunoglobulins—combined with severe energy and macronutrient restriction that impairs cellular, systemic, and mucosal immune defenses, constitute the primary mechanisms underlying increased infection susceptibility [10,21].

Extending these observations to cellular immunity, Shimizu et al. [13] examined the effects of a 2-week weight loss program on T-cell and monocyte populations in 6 elite male judo athletes (20.3 ± 0.4 years). Flow cytometry analysis revealed significant reductions in multiple lymphocyte subpopulations during the weight loss period compared to baseline: CD3^+^, CD4^+^, CD8^+^, and CD28^+^CD4^+^ cells all declined substantially. These cellular deficits reverted to baseline values 1 day after competition, suggesting reversibility of the immunosuppressive effects. Notably, Toll-like receptor (TLR)-4-positive CD14^+^ monocytes, which mediate innate immune responses to bacterial pathogens, decreased significantly during weight loss and remained suppressed even after competition, indicating prolonged impairment of innate immune surveillance. Consistent with these cellular immune deficits, athletes reported URTI symptoms during the weight loss period, including 1 headache, 3 instances of runny nose, and 1 episode of coughing. The authors proposed that the suppression of T-cell subpopulations compromises cell-mediated adaptive immunity, while the persistent reduction in TLR-4-expressing monocytes impairs pathogen recognition and innate immune activation, collectively rendering athletes highly susceptible to opportunistic infections [13,22].

The mechanistic basis for RWL-induced immunosuppression emerges from the convergence of multiple physiological stressors. Severe caloric restriction (40–50% reduction in energy intake) depletes muscle and hepatic glycogen stores, which triggers IL-6 release from skeletal muscle as an adaptive metabolic response [4]. Concomitant dehydration, evidenced by body water losses exceeding 10%, concentrates plasma proteins while reducing mucosal secretions, thereby impairing the physical and immunological barriers of the respiratory and gastrointestinal mucosa [12]. The hormonal milieu shifts toward a catabolic, immunosuppressive state characterized by hypercortisolemia and hypotestosteronemia, which collectively suppress lymphocyte proliferation, cytokine production, and NK cell activity [4]. Intensified training loads during the weight-cutting period further deplete immune cell populations and reduce immunoglobulin synthesis, creating an open window of heightened infection vulnerability [10]. Notably, the observed increase in neutrophil phagocytic activity during weight loss, while seemingly paradoxical, may reflect a compensatory response to decreased circulating neutrophil numbers or altered cellular activation states rather than enhanced immune capacity [10]. The persistence of reduced TLR-4-expressing monocytes even after weight recovery suggests that certain innate immune defects may require extended recovery periods, potentially leaving athletes vulnerable during the critical peri-competition phase [13].

The experimental evidence demonstrates that RWL in combat sports athletes precipitates multifaceted immunological dysfunction encompassing mucosal immunity suppression, systemic inflammatory dysregulation, cellular immune deficits, and hormonal perturbations [4,5,6,9,10,13,14]. These alterations occur through mechanisms independent of plasma glutamine status and manifest clinically as increased incidence and severity of respiratory infections [10,23]. The magnitude of weight loss emerges as a critical determinant, with reductions exceeding 5% of body weight producing particularly severe immunological consequences. These findings underscore the necessity for combat sport athletes, coaches, and medical personnel to implement more gradual, physiologically sustainable weight management strategies are paramount to balancing competitive demands with immunological integrity (Table 2, Table 3 and Table 4).

## 4. Discussion

### 4.1. Hypothalamic–Pituitary–Adrenal Axis Activation and Immune Function

Within hours of initiating severe caloric restriction and dehydration, the hypothalamus secretes corticotropin-releasing hormone, which stimulates adrenocorticotropic hormone (ACTH) release from the anterior pituitary and drives a 200–300% elevation in plasma cortisol within 24 h [31]. These glucocorticoid surges, together with concomitant sympathetic activation of epinephrine and norepinephrine, orchestrate widespread immunomodulation. Cortisol induces rapid lymphocyte redistribution, causing a 20–40% lymphopenia by 24–48 h post-RWL and impairing T-cell-mediated responses through reductions of 30–50% in IL-2 and interferon (IFN)-γ synthesis upon mitogen challenge [3]. At the molecular level, these effects are mediated through distinct genomic pathways. Cortisol binds to intracellular glucocorticoid receptors, creating a complex that translocates to the nucleus and directly inhibits the transcriptional activity of NF-κB and Activator Protein-1 [32,33]. This blockade profoundly suppresses the synthesis of IL-2, the critical growth factor for T-cell proliferation and clonal expansion [3]. Concurrently, testosterone acts via androgen receptors to further modulate this landscape; while physiological levels dampen excessive pro-inflammatory Th1 responses, the sharp decline in testosterone during RWL disrupts this delicate balance, potentially impairing the regulatory feedback loops that control cytokine production [19]. Meanwhile, innate immune cells are paradoxically mobilized yet functionally suppressed: neutrophil demarginating yields a 30–50% increase in circulating counts, but nicotinamide adenine dinucleotide phosphate (NADPH) oxidase inhibition and adenosine triphosphate (ATP) depletion underlie 20–35% declines in both phagocytic capacity and oxidative burst activity [34]. The NK cell cytotoxicity similarly falls by up to 25% at 24 h post-RWL, reflecting glucocorticoid-driven downregulation of activating receptors and diminished IL-2 support [35,36]. Cortisol exhibits context-dependent effects on transforming growth factor (TGF)-β signaling pathways [37], with dose- and duration-dependent modulation of FoxP3^+^ regulatory T cell function that can range from enhancement under acute physiological conditions to impairment under chronic stress exposure [32]. While cortisol suppresses IL-10 production in regulatory B cells through miR-98 upregulation [38], its effects on Treg-derived IL-10 are complex, as IL-10 signaling in Treg cells creates positive feedback loops through signal transducer and activator of transcription 3 activation that may actually enhance IL-10 production under certain conditions [33]. The resulting deficit in immunoregulatory cytokines prolongs proinflammatory cascades and extends the post-competition “open window” of immunosuppression, during which upper respiratory tract infection risk rises by up to 30% [27]. Interestingly, while systemic immunoglobulins (e.g., serum IgG, IgM) often remain stable or appear transiently elevated due to hemoconcentration, sIgA exhibits a distinct and significant reduction following RWL. This selective suppression of mucosal immunity is mechanistically driven by two primary factors. First, elevated cortisol levels downregulate the expression of the polymeric immunoglobulin receptor on mucosal epithelial cells, thereby impairing the intracellular transport of IgA from the lamina propria to the lumen [26]. Second, severe dehydration reduces saliva flow rate, limiting the delivery of sIgA to the mucosal surface despite potentially preserved B-cell synthesis rates [12]. Consequently, RWL specifically compromises the “first line of defense” in the upper respiratory tract without necessarily indicating systemic B-cell failure. These neuroendocrine and immunological perturbations manifest clinically as delayed recovery, increased illness incidence, and compromised performance in weight-categorical competition. To mitigate HPA-mediated immunodeficiency, graded weight-management strategies are essential. Limiting weight loss to ≤1% of body mass per week substantially attenuates cortisol and catecholamine surges. Nutritional interventions ensuring energy intake ≥30 kcal·kg^−1^·day^−1^ with 1.2–1.7 g·kg^−1^·day^−1^ protein support adrenal homeostasis and preserve immune cell bioenergetics [39]. Supplementation with omega-3 fatty acids and vitamin D has been shown to enhance IL-10 and TGF-β pathways, facilitating Treg recovery and restoring cytokine balance [40,41]. Furthermore, it is crucial to recognize that the immunological consequences of RWL are not limited to acute perturbations but can accumulate over time. Repeated cycles of RWL, often referred to as weight cycling, contribute to chronic immune dysregulation through the accretion of “Allostatic load”. Frequent and intense activation of the HPA axis can lead to glucocorticoid receptor resistance, resulting in a failure to downregulate inflammatory cytokines (e.g., IL-6, TNF-α) efficiently even during rest periods [4]. Moreover, the recurrent oxidative stress associated with rapid dehydration and subsequent rehydration phases depletes endogenous antioxidant reserves, such as glutathione and superoxide dismutase [7]. This cumulative depletion creates a state of chronic systemic oxidative stress that impairs lymphocyte viability and function long-term. While a specific threshold number of cycles has not been universally defined, evidence suggests that athletes engaging in frequent RWL (e.g., >3–4 times per year) are at a significantly higher risk of blunted immune responsiveness compared to their weight-stable counterparts [1,2].

### 4.2. Neutrophils and Rapid Weight Loss

Although peripheral neutrophil counts rise by 30–50% post-RWL, cell-based assays consistently report 20–35% decreases in phagocytic capacity and 25–40% reductions in oxidative burst intensity [24]. This quantitative–qualitative disconnect is mechanistically rooted in the convergent stresses of severe caloric restriction and dehydration, which hyperactivate the HPA axis, precipitating surges of cortisol and catecholamines [42]. These hormones drive neutrophil demarginating and bone-marrow egress while simultaneously disrupting endothelial adhesion, impairing phagosome biogenesis, and inhibiting ROS generation [43,44]. Compounding these neuroendocrine effects, energy deficits deplete glycolytic ATP reserves essential for phagocytic processes, protein insufficiency compromises assembly of the NADPH oxidase complex, and micronutrient shortfalls—particularly of iron and zinc—erode enzymatic cofactor availability and redox buffering capacity [44]. The resulting state of neutrophil dysfunction perpetuates low-grade systemic inflammation, as evidenced by concurrent elevations in TNF-α, IL-1β, IL-8, and lipid peroxidation markers such as malondialdehyde [45,46]. Despite a transient increase in circulating neutrophil numbers—a phenomenon known as demarginating—functional capacity is diminished. This paradox is well-documented in experimental studies. For example, Suzuki et al. [24] investigated female judo athletes undergoing a 5% body mass reduction over three days. Blood samples collected before, immediately after, and eight days post-intervention revealed a significant decrease in neutrophil phagocytic activity (approximately 28% reduction, *p* < 0.01) compared to controls. While oxidative burst activity increased in both groups due to exercise, the decrease in phagocytic function was more pronounced in the weight reduction group, suggesting that energy restriction and dehydration, coupled with elevated cortisol, impair neutrophil metabolism and function. Also, physique athletes assigned to RWL showed marked increases in total white blood cells and leukocyte counts, significantly increased normal ranges [25]. In contrast, the immune variables in the control group remained in the normal range. Despite the increase in cell numbers, functional impairment and increased risk of URTI were observed in the RWL group, highlighting the disconnect between quantity and quality of complex immune dysregulation [25]. Some studies report that RWL may not significantly alter ROS generation by neutrophils, or may even transiently increase it due to exercise stress [47,48]. However, the overall trend is a reduction in phagocytic capacity and increased infection risk, suggesting that other components of the immune system, such as mucosal immunity and adaptive responses, are also compromised. Clinically, the functional decline in neutrophil activity following RWL has direct consequences for athlete health and performance. Increased susceptibility to infections, delayed recovery from muscle damage, and prolonged inflammation are commonly reported during and after weight-cutting periods [49,50]. These outcomes are particularly concerning in combat sports, where athletes are exposed to high physical contact and environmental stressors that can further challenge immune resilience [49,50]. The evidence suggests that protecting neutrophil function through gradual weight management, adequate nutrition, and hydration is essential for maintaining immune competence and optimizing performance in combat sports athletes. Collectively, RWL induces complex changes in neutrophil biology, characterized by transient increases in cell numbers but persistent declines in functional capacity. These alterations are driven by cortisol-mediated metabolic shifts and are exacerbated by dehydration and nutrient deficiencies, highlighting the vulnerability of the innate immune system to metabolic stress.

### 4.3. Monocyte and Macrophage System Alterations

The physiological stress of RWL—driven by severe caloric restriction, dehydration, and intense exercise—triggers a cascade of neuroendocrine and metabolic responses that directly impact monocyte dynamics, subset distribution, and macrophage polarization. Monocytes and their tissue-resident derivatives, macrophages, function as critical arbiters of inflammation resolution and tissue repair, yet RWL in athletes causes a maladaptive reprogramming of this axis. Acute RWL elicits a 25–40% rise in circulating monocyte counts, primarily expanding classical (CD14^++^CD16^−^) and intermediate (CD14^+^CD16^+^) subsets. Crucially, this expansion is driven by physiological stress and metabolic endotoxemia rather than a pre-existing active infection [43,44]. These mobilized monocytes exhibit a skewed pro-inflammatory phenotype, characterized by elevated basal secretion of IL-1β, IL-6, and TNF-α [28,29,30]. However, this hyper-inflammatory state does not translate into enhanced host defense; instead, it represents a dysregulated immune response. While their cytokine output is elevated, their specific antimicrobial functions—namely, pathogen recognition via TLR-4 and subsequent phagocytic activity—are significantly impaired [13,22]. This functional dissociation, therefore, renders the athlete vulnerable to infection despite the apparent increase in circulating immune cells and inflammatory markers. Mechanistically, severe caloric restriction and dehydration hyperactivate the HPA axis, triggering cortisol and catecholamine surges that mobilize bone marrow precursors and prime monocytes via NF-κB–dependent pathways [30]. Concurrent suppression of nonclassical, patrolling monocytes skews the circulating pool toward inflammation-propagating phenotypes. Upon tissue-infiltration, these primed monocytes differentiate into M1-dominant macrophages—with heightened CD80/CD86 expression and sustained IL-6/TNF-α production—that perpetuate low-grade systemic inflammation and impede myofiber regeneration [51,52]. In contrast, gradual weight-loss protocols (≤1% body mass per week) limit monocytosis (<10%) and favor enrichment of nonclassical (CD14^+^CD16^++^) monocytes; macrophage polarization shifts toward an M2 phenotype, marked by CD206 upregulation and enhanced IL-10 and TNF-β secretion, which promote inflammation resolution and tissue-repair [53,54]. Clinically, RWL-associated monocyte–macrophage dysregulation correlates with prolonged muscle soreness, delayed recovery between bouts, and increased upper respiratory tract infection incidence, thereby undermining both athlete health and competitive readiness. In a controlled trial by Tritto et al. [10], combat athletes subjected to RWL protocols exhibited significant changes in immune cell function, including fluctuations in monocyte phagocytic activity. While the magnitude of change in monocyte phagocytosis was modest, the study reported an increased frequency and severity of infection symptoms during and after weight reduction, suggesting that functional alterations in monocytes and their downstream macrophage derivatives may contribute to immunosuppression. Notably, the negative effects of RWL on cell-mediated immunity were attributed to restricted energy intake, increased exercise volume, and chronic hypohydration, all of which are common in combat sports preparation [10,14]. Animal studies provide additional insight into the dynamics of macrophage recruitment and polarization during weight loss. For instance, research in obese mice undergoing caloric restriction revealed a transient increase in adipose tissue-macrophages (ATMs) during the initial phase of weight loss, followed by a progressive decrease with sustained restriction [55]. Interestingly, the early influx of macrophages did not correspond to a marked change in inflammatory gene expression, suggesting that the functional phenotype of these cells may be context-dependent and influenced by the duration and severity of weight loss [56]. In summary, RWL in combat sports athletes leads to quantitative and qualitative alterations in the monocyte–macrophage system. These changes are mediated by neuroendocrine stress responses and are characterized by increased proinflammatory monocyte subsets, enhanced M1 macrophage polarization, and elevated production of inflammatory cytokines. Such alterations may compromise immune defense, delay recovery, and increase susceptibility to infection, highlighting the need for evidence-based weight management strategies that preserve immune function while supporting athletic performance.

### 4.4. Changes in NK Cell Activity and Functionality of T Cell Subpopulations

NK cells and T lymphocyte subpopulations constitute the cornerstone of cell-mediated immunity, yet RWL in athletes precipitates a profound dysfunction across these critical effector arms. NK cells, which eliminate virally infected or transformed cells through perforin- and granzyme-mediated cytotoxicity, are critically dependent on glycolytic ATP generation and amino acid substrates; severe caloric restriction and dehydration inherent in RWL protocols deplete glucose, glutamine, and essential amino acids, thereby impairing cytolytic granule synthesis and exocytosis [57,58]. Concurrent hyperactivation of the HPA axis elevates circulating cortisol and catecholamines, which not only suppress NK cell proliferation but also downregulate activating receptors such as natural killer group 2, member D (NKG2D), compounding functional deficits despite occasional transient rebounds in circulating NK counts that represent stress-induced mobilization rather than restored competence [59]. Simultaneously, energy and neuroendocrine stressors attenuate IL-2 production—vital for NK cell maturation—and disrupt the balance of CD4^+^ T helper cell polarization [59,60]. While evidence from studies in other sports and animal models suggests that RWL may suppress Th1 activity and relatively upregulate Th2 and Th17 subsets direct confirmation in combat sports athletes remains limited. Therefore, such immunological shifts should be interpreted cautiously and further validated in combat sports-specific populations. Animal and in vitro research indicate a potential decline in FoxP3+ regulatory T cell frequency during rapid energy deficiency [61,62], but direct evidence for this phenomenon following RWL in elite combat sports athletes is not yet established and should be further investigated. In a study by Oh et al. [63], physical exercise and vitamin D status were identified as major determinants of NK cell activity, with intense training and nutritional deficits during RWL leading to reduced NK cell function. Similarly, Barra et al. [64] found that high-intensity interval training could increase NK cell number and activity, but the benefits were blunted in the context of energy restriction and dehydration typical of RWL protocols. This reduction in NK cell activity is further exacerbated by dehydration and micronutrient deficiencies, which impair cellular metabolism and signaling pathways. T cell subpopulations are also affected by RWL. Cortisol-induced lymphopenia leads to a decrease in both CD4^+^ helper and CD8^+^ cytotoxic T cells, with studies showing reductions in circulating during the acute phase of RWL [65]. Moreover, there is evidence of a Th1-to-Th2 shift, with Th1 cytokines such as IFN-γ decreasing and Th2 markers like IL-4 increasing [66]. These changes compromise both innate and adaptive immune defenses, increasing the risk of infection and impairing recovery. Prioritizing conservative weight management practices alongside comprehensive hydration and dietary planning is paramount to safeguarding cytotoxic immune function [67]. Despite the combined knowledge, direct profiling of T cell subsets in combat athletes remains limited, and further research is needed to clarify consequences of RWL on immunity [50].

### 4.5. Acute-Phase Response of Complement Systems and Changes in Inflammatory Cytokines

RWL in combat sports athletes triggers acute energy deficit and dehydration, leading to hyperactivation of the HPA axis and a systemic acute-phase response (APR). This process induces dramatic changes in hepatic synthesis of major acute-phase proteins (APPs), including complement components C3 and C4, and C-reactive protein (CRP), while reducing the production of negative APPs such as albumin and transferrin [68,69,70,71]. Within hours of severe caloric restriction and dehydration, activation of the HPA axis drives IL-6 elevations of 233% by post-RWL [68], sustaining above-baseline concentrations for up to 48 h and prompting hepatocyte synthesis of positive APPs including C-reactive protein (CRP) and complement components C3 and C4 [68,69]. CRP levels surge 2.5 to 19-fold within 24–48 h, enhancing pathogen opsonization but also reflecting substantial APR engagement while complement C3 and C4 mirror this early rise [68,69,70,71], before paradoxically declining under extended energy deficits beyond 72 h—an indication of hepatic resource reallocation that compromises complement-mediated microbial clearance [72]. Concurrent spikes in IL-1β and TNF-α, peaking at 24 h and 72 h, respectively, further amplify leukocyte recruitment and endothelial activation, thereby extending the “open-window” of post-competition immunosuppression [26]. Serial profiling at 24, 48, and 72 h reveals distinct cytokine kinetics: While IL-1β and TNF-α regress toward baseline by 72 h when aggressive refeeding and rehydration are instituted, the absence of such nutritional recovery allows IL-6 and CRP to remain elevated, thus increasing URTI risk by up to 30% during competitive phases [26,73]. This protracted APR not only undermines mucosal immunity and pathogen clearance but also delays myofiber repair and prolongs recovery timelines—critical impediments for athletes requiring rapid bout turnover [71]. Moreover, sustained APR engagement fosters insulin resistance, proteolysis, and metabolic inflexibility, compounding performance decrements and elevating injury risk [71]. In a related study, demonstrate that aggressive weight reduction strategies—such as increased training loads and severe caloric restriction—can lead to transient increases in complement components like C3 prior to competition, followed by significant decreases in immunoglobulins (IgG, IgM) and complement proteins (C3) in the days after competition. Jung et al. [74] found that judo athletes who underwent RWL experienced a marked reduction in these immune markers seven days post-competition, with the effect being more pronounced in those who combined energy restriction with intense exercise. These findings suggest that repeated cycles of RWL may cumulatively induce abnormal immune marker levels, potentially increasing clinical risk for athletes. In addition, RWL is associated with increased symptoms of infection and immunosuppression, likely due to altered cytokine responses. Studies have reported that athletes undergoing RWL show elevated levels of pro-inflammatory cytokines such as IL-6 and TNF-α, which are known to drive acute-phase protein synthesis and amplify systemic inflammation. Although some interventions, like glutamine supplementation, have been tested to mitigate these effects, they have not consistently prevented the increased frequency of infection symptoms observed during and after RWL [10]. Overall, these experimental results highlight that RWL disrupts immune homeostasis by modulating both complement proteins and inflammatory cytokines. The initial phase of RWL may trigger transient immune activation, but prolonged or repeated RWL cycles can result in immunosuppression, increased infection risk, and abnormal inflammatory responses. These findings underscore the importance of monitoring immune markers and adopting safer, phased weight loss strategies to protect athlete health.

### 4.6. Changes in B-Cell and Antibody Production and Anti-Inflammatory Cytokine Responses

**Figure 3:**
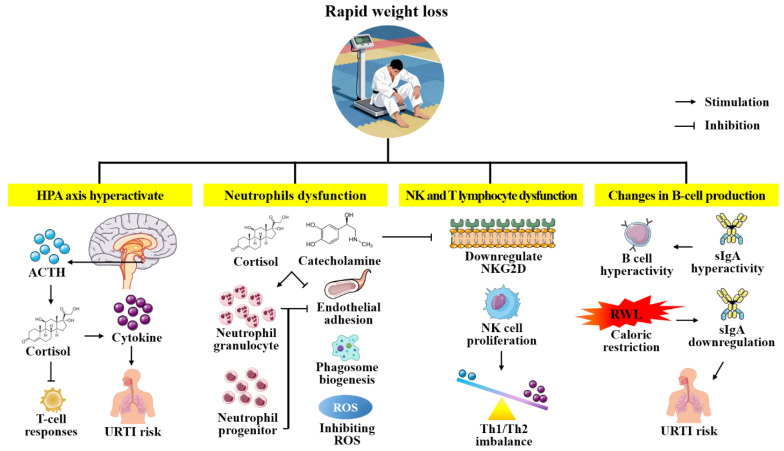
Schematic of the effects of rapid weight loss on immune system. Severe caloric restriction, dehydration, and intensive training activate the HPA axis, elevating cortisol and suppressing both innate and adaptive immunity. Key alterations include: reduced lymphocyte proliferation and NK cell cytotoxicity; impaired neutrophil phagocytic function despite increased counts; proinflammatory monocyte/macrophage polarization; decreased mucosal sIgA secretion; and sustained acute-phase response with elevated IL-6, TNF-α, CRP, and complement proteins. These changes create an extended post-competition immunosuppression window, increasing URTI risk and delaying recovery. HPA: hypothalamic–pituitary–adrenal; ACTH: adrenocorticotropic hormone; URTI: upper respiratory tract infections; ROS: NK: natural killer; ROS: reactive oxygen species; NKG2D: natural killer group 2, member D; RWL: rapid weight loss.

B cells and their antibody products constitute the backbone of humoral immunity, yet in combat sports athletes undergoing RWL this critical arm of host defense undergoes a biphasic perturbation characterized by transient activation followed by sustained suppression, with direct implications for infection susceptibility and recovery dynamics. Initially, acute neuroendocrine stress triggered by severe caloric restriction and dehydration induces elevation in sIgA reflecting adrenal-mediated upregulation of mucosal B cell trafficking and exocytosis [12]. Mechanistically, this transient upregulation is driven by catecholamines (epinephrine and norepinephrine) binding to β2-adrenergic receptors (β2-AR) expressed on B cells [12,42,75]. Activation of β2-AR signaling enhances chemokine receptor responsiveness, facilitating the rapid mobilization of B cells from marginal pools to mucosal effector sites. Furthermore, adrenergic stimulation activates the intracellular cAMP-protein kinase A pathway, which directly promotes the fusion of IgA-containing vesicles with the plasma membrane, thereby accelerating secretory exocytosis [12,76]. However, this early surge is rapidly eclipsed by a 30–45% decline in sIgA concentrations over the subsequent 48–72 h as energy and amino acid substrates become limiting, compromising B cell proliferation, class-switch recombination, and secretory machinery. The decrement in sIgA correlates closely with a two- to three-fold increase in URTI reported during training camps and competition phases, underscoring the functional consequences of mucosal antibody depletion [12]. Sarin et al. [25] reported that athletes undergoing RWL showed a marked increase in total white blood cell count, primarily due to elevated neutrophil numbers, while the relative percentage of lymphocytes—including B cells—decreased. These changes were accompanied by reductions in erythrocyte and platelet counts, suggesting that RWL affects not only immune cell populations but also broader hematopoietic processes. Importantly, these alterations were reversible, as blood cell counts returned to baseline after weight regain, indicating a dynamic but potentially risky shift in immune homeostasis during RWL periods. Meanwhile, anti-inflammatory cytokines, such as IL-10 and TGF-β, play a crucial role in regulating immune responses and preventing excessive inflammation. Evidence suggests that RWL can disrupt the balance between pro- and anti-inflammatory cytokines [25,77,78]. The cumulative evidence indicates that RWL disrupts B-cell homeostasis, reduces antibody production, and alters cytokine responses, increasing susceptibility to infections and impairing recovery (Figure 3). Accordingly, athletes practicing RWL should be monitored for changes in immunoglobulin levels and cytokine profiles, and advised to implement phased weight reduction strategies supported by targeted nutrient timing to preserve humoral immunity. Interventions targeting, stress management, and anti-inflammatory nutrition may help mitigate some of the adverse effects, but further research is needed to establish effective strategies [10,25,77,78].

### 4.7. Practical Implications and Evidence-Based Strategies

Integrated intervention strategies for mitigating the negative effects of RWL in combat sports athletes should be grounded in a comprehensive understanding of the physiological mechanisms involved. RWL imposes multifaceted stress on the athlete’s body, particularly affecting the neuroendocrine and immune systems, substrate availability, and neuromuscular function. Therefore, a successful intervention must address each of these axes in a coordinated and evidence-based manner.

The first pillar of an integrated approach is the periodization of the weight-cutting process. Rather than resorting to abrupt and severe caloric restriction, evidence suggests that athletes should adopt a structured plan that gradually reduces body mass over a period of at least two weeks. Empirical studies indicate that limiting weight loss to approximately 1% of body mass per week may attenuate the neuroendocrine stress response, as evidenced by lower circulating cortisol and catecholamine levels, and helps preserve immune cell function compared to more aggressive protocols. This gradual approach also appears to reduce the risk of lymphocyte apoptosis and neutrophil dysfunction, which are commonly observed in rapid, unstructured weight cuts [79]. During the pre-competition phase, a moderate caloric deficit of 3–10% below maintenance, combined with the maintenance of training volume, can prevent performance decrements while promoting fat loss [80]. In the final days before competition, shifting focus to smaller energy deficits and the repletion of muscle glycogen may support mucosal immune recovery and the normalization of sIgA levels [81]. After competition, the priority should be full rehydration, carbohydrate restoration, and antioxidant replenishment to expedite the normalization of cytokine profiles and complement activity, thereby reducing the duration of immunosuppression and the risk of upper respiratory tract infections [82,83,84]. However, it should be noted that the feasibility of such gradual protocols depends on the athlete’s starting body composition and competition schedule, and individual responses to tapering may vary.

Nutritional optimization is the second essential component. During periods of weight loss, maintaining total energy intake above 10 kcal/kg/day is recommended, as more severe deficits are associated with declines in lymphocyte proliferation and NK cell cytotoxicity [85]. Protein intake should ideally be maintained at 1.4–2.0 g/kg/day to support immunoglobulin synthesis and inhibit muscle proteolysis [86]. Randomized trials in combat athletes have demonstrated that higher protein intakes help preserve post-exercise immune function and muscle strength compared to lower protein regimens [87,88]. Additionally, ensuring carbohydrate intake of 5–7 g/kg/day can sustain neutrophil and NK cell glycolysis, which is essential for maintaining oxidative burst capacity [89]. Supplements with omega-3 fatty acids (3 g EPA/DHA daily) [90,91] and vitamin D_3_ (2000 IU/day) [92,93] has been shown to potentially upregulate anti-inflammatory cytokines and enhance regulatory T cell frequency [94]. In addition, micronutrient and antioxidant support is important because RWL increases the production of ROS, which can impair muscle contractility and immune cell viability [95]. Furthermore, micronutrient support, including vitamin C (6 g/day) [96], vitamin E (<500 IU/day) [97], selenium (1000–1600 µg/day) [98] and zinc (30–50 mg/day) [99] may help mitigate the increased production of ROS and maintain redox balance.

Hydration strategies must be carefully periodized to match the different phases of weight management. During the pre-competition taper, consuming sufficient fluid (e.g., 26 mL/kg) [100] with adequate sodium (about 3.2 g/L) [101] may help maintain plasma volume and support renal clearance of oxidative metabolites. Acute rehydration following weigh-in should involve hypertonic saline and isotonic fluids to rapidly restore intravascular volume. Training and recovery modulation is another key aspect. Reducing training intensity by approximately 30% during active weight-cut phases, with an emphasis on technical skill work, may limit additional metabolic stress [102]. Post-competition recovery protocols, including cold-water immersion, compression therapy, and extended sleep, have been shown to accelerate cytokine normalization and muscle repair [103,104,105,106]. However, the optimal rehydration strategy can vary depending on the extent of dehydration and individual sweat rates, necessitating a tailored approach.

Finally, longitudinal biomarker monitoring enables data-driven personalization of intervention strategies. Regular assessment of salivary cortisol, sIgA, plasma IL-6, CRP, CD4/CD8 ratios, neutrophil oxidative burst, and regulatory T cell frequency allows for the early detection of adverse trends. When biomarkers fall outside of established thresholds, protocol adjustments such as pausing weight cuts, increasing caloric intake, or delaying high-intensity training should be considered to ensure immunological resilience and optimal performance [107,108]. Like this, an integrated intervention strategy for RWL in combat sports athletes must be multifaceted, addressing the physiological, nutritional, and training-related challenges posed by rapid weight reduction. By grounding each component in scientific evidence and mechanism understanding, it is possible to mitigate the negative effects of RWL and support both the health and performance of athletes. While monitoring offers significant advantages, its practical application may be limited by cost, accessibility of testing equipment, and the need for expert interpretation.

### 4.8. Integrated Recommendations for Coaches and Athletes: Weight Loss Strategy

- Rate of Loss: Coaches and athletes should strictly adhere to a weekly weight loss rate of less than 1% of total body mass. This gradual approach allows for metabolic and immune adaptation, minimizing the catabolic stress (HPA axis activation) typically seen in RWL.
- Critical Threshold: Immunological risks escalate significantly when athletes target a body mass reduction exceeding 5% in the final 72 h before competition. Protocols that necessitate such acute reductions should be avoided entirely due to the documented severe decline in lymphocyte function and sIgA levels.
- Hydration: While mild dehydration is common, severe water restriction should be minimized. Fluid intake should be closely monitored using urine specific gravity to ensure hydration status remains within safe clinical thresholds, especially in the final days leading up to the weigh-in.

### 4.9. Integrated Recommendations for Coaches and Athletes: Nutrition Intake Strategy

Nutritional intervention must prioritize adequate substrate availability to fuel immune cells and maintain gut barrier integrity.

- Carbohydrate Intake: Even during energy restriction, the protocol should maintain a minimal carbohydrate intake (5 g/kg/day) to protect glycogen stores and prevent excessive cortisol elevation [89]. Furthermore, the 48-h post-weigh-in period is crucial for immune recovery; carbohydrate loading (e.g., 8–10 g/kg/day) should be initiated immediately after weigh-in to accelerate glycogen and immune cell recovery.
- Protein and Micronutrients: Consistent protein intake (1.4–2.0 g/kg/day) is essential to prevent lean mass loss and provide the necessary amino acids (e.g., BCAA’s) for immune cell synthesis [86]. Furthermore, attention must be paid to micronutrients critical for immune function, including Vitamins C, E, and Zinc, which should be supplemented if dietary intake is compromised [96,97,99].

### 4.10. Integrated Recommendations for Coaches and Athletes: Real-Time Feedback

To provide objective, real-time feedback on immune status, simple and non-invasive biomarkers should be regularly tracked.

- Primary Biomarkers: sIgA and salivary cortisol are cost-effective, non-invasive indicators of mucosal immune integrity and HPA axis activity, respectively. A significant decrease in sIgA concentration or a sustained elevation in resting cortisol levels should signal a mandatory halt or severe adjustment of the weight loss protocol.
- Clinical Oversight: Coaches and athletic trainers should collaborate with a sports physician or dietitian to periodically assess complete blood count CBC to monitor lymphocyte and neutrophil counts and interpret these biomarker results within the context of the athlete’s training load and diet.

### 4.11. Integrated Recommendations: Author-Derived Guidelines with Limitations

The integrated recommendations presented above (Section 4.8, Section 4.9 and Section 4.10) were derived by the author through comprehensive synthesis of the 10 included combat sports-specific studies and supporting mechanistic literature reviewed herein. These guidelines integrate empirical findings on RWL-induced immune perturbations with established sports immunology principles to provide practical, evidence-informed strategies. However, implementation should account for key limitations of the current evidence base, including: (1) small sample sizes (*n* < 30 in 80% of studies), (2) heterogeneity in RWL protocols (3–10% BW loss over 3–14 days), (3) lack of long-term immunological outcomes beyond 1–2 weeks post-RWL, and (4) absence of randomized controlled trials directly testing intervention efficacy in elite athletes. Individual athlete monitoring (e.g., sIgA, cortisol levels) and consultation with sports medicine specialists are strongly recommended prior to application.

### 4.12. Limitations and Future Directions

Despite the insights provided in this review, several limitations must be acknowledged. First, the review protocol was not prospectively registered, which may introduce potential reporting bias. Second, the majority of included studies were short-term and focused primarily on elite athletes, limiting the generalizability of findings to female athletes, youth populations, or club-level practitioners. Third, the heterogeneity of RWL protocols (varying in duration and magnitude) and the lack of standardized immune outcome measures complicate direct comparisons across studies. Future research should prioritize longitudinal studies that examine the cumulative effects of repeated weight cycling on immune competence and investigate demographic characteristics-specific immune responses to RWL. Additionally, more rigorous randomized controlled trials are needed to validate the efficacy of specific nutritional and recovery interventions in this unique population.

## 5. Conclusions

This systematic review demonstrates that RWL in combat sports athletes induces multifaceted immunological dysfunction, characterized by the suppression of both innate and adaptive defense mechanisms. Specifically, RWL involving severe caloric restriction and dehydration activates the HPA axis, leading to cortisol-mediated lymphopenia, impaired NK cell cytotoxicity, and reduced neutrophil function. Furthermore, the decline in mucosal immunity, evidenced by decreased sIgA levels, significantly elevates the risk of upper respiratory tract infections during the critical pre-competition window. The magnitude of weight loss serves as a key determinant of immune compromise, with reductions exceeding 5% of body mass producing the most severe perturbations. Consequently, traditional RWL practices not only jeopardize athlete health but may also undermine competitive readiness through delayed recovery and increased illness susceptibility. To mitigate these adverse effects, an integrated approach combining gradual weight management (<1% per week), nutritional optimization, and strategic periodization is essential. Coaches and medical staff should prioritize evidence-based strategies that balance weight category requirements with the preservation of immunological integrity, ultimately safeguarding the long-term health and performance of combat sports athletes.

## Figures and Tables

**Table 1:** Full search strategies used for each database.

Database	Search Query String
PubMed	(“rapid weight loss” OR “weight reduction” OR “weight cutting” OR “weight loss”) AND (“combat sports” OR “taekwondo” OR “judo” OR “wrestling” OR “boxing”) AND (“immune system” OR “immune response” OR “cytokines” OR “inflammation” OR “sIgA” OR “natural killer cells”)
Web of Science	(“rapid weight loss” OR “weight reduction” OR “weight cutting”) AND (“combat sports” OR “taekwondo” OR “judo” OR “wrestling”) AND (“immune system” OR “immune response” OR “cytokines” OR “inflammation” OR “sIgA”)
Scopus	(“rapid weight loss” OR “weight reduction”) AND (“combat sports” OR “taekwondo” OR “judo”) AND (“immune system” OR “cytokines” OR “sIgA”)

**Table 2:** Studies on the effects of rapid weight loss on the immune system.

Study	Participants	RWL Intervention	Measures	Results	Quality
Abedelmalek et al. 2015 [4]	11 male Judo athletes (20.45 ± 0.51 years)	7-day caloric restriction (−6 MJ/day) - Only one glass of water (15 to 20 cL)	- BMI * - HR ** - Performance - Blood collection	- BW *** ↓ - Performance ↓ - TNF-α ↑ - IL-6 ↑ - Cortisol ↑ - GH **** ↑ - Testosterone ↓	NOS Score: 8/9
Shimizu et al. 2011 [13]	6 male Judo athletes (20.3 ± 0.4 years)	Self-determined weight loss programs (dietary energy restriction, fluid restriction, bicycle exercise in the dry room, wearing sauna suits during training, and sauna)	- BMI - Blood collection - URTI symptoms	- CD3 ↓ - CD4 ↓ - CD8 ↓ - CD28CD4 ↓ - TLR-4CD14 ↓ - URTI symptoms ↑	NOS Score: 7/9
Kaya et al. 2016 [14]	10 male Taekwondo athletes (20.67 ± 0.24 years)	4-week Taekwondo exercise program + exhaustion exercise (Bruce protocol)	- Blood collection - ELISA *****	- IFN-γ – - TNF-α – - IL-2 ↑ - IL-6 ↓	NOS Score: 7/9
Lee et al. 2012 [6]	6 female Taekwondo athletes (16.07 ± 0.8 years)	5 bouts of Taekwondo competitions (maximal heart rate 92.2 ± 3.8%)	- Blood collection - Flow-cytometry	- NK cells ↑ - B cells ↑ - T cells ↑ - CD4/CD8 ratio ↓ - Lactate ↑ - ROS ^#^ ↑	NOS Score: 8/9
Tsai et al. 2011a [5]	16 male Taekwondo athletes (21.6 ± 1.3 years)	7-week training, competition, and recovery period → included RWL immediately 1-week pre-competition	- Body composition - Blood collection - Saliva sampling - URTI incidence	- BW ↓ - sIgA ↑ - Cortisol – - URTI incidence ↑	NOS Score: 8/9
Yang et al. 2015 [15]	10 male Taekwondo athletes (21.1 ± 5.48 years)	Group A: 5% BW reduction within 4 days vs. Group B: 5% BW reduction within 4 weeks	- Body composition - Blood collection - Immunohistochemical staining	*Compared to Group B*, - RBC ^##^-NO ^###^ activation ↓ - RBC Nitrite/NO ↓ - RD ^####^ ↓ - RA ^#####^ ↑ - DT ^######^ ↑	NOS Score: 8/9
Tsai et al. 2011b [9]	10 female Taekwondo athletes (21.3 ± 1.2 years)	Intense Taekwondo training + Group A: RWL vs. Group B: non-RWL	- Saliva sampling	*Compared to Group B*, - sIgA ↓ - Cortisol ↑ *Compared to Group A*, - Lactoferrin ↓	Low Risk of Bias
Tritto et al. 2018 [10]	Mixed combat sports (judo, wrestling, taekwondo)/Group A: 23 male athletes, Group B: 6 male athletes (20 ± 2 years)	Group A: 5–10% BW reduction within 21 days + supplementation glutamine vs. Group B: 5–10% BW reduction within 21 days + supplementation placebo	- Body composition - Blood collection - URTI symptoms	- Body composition – *Compared to Group B*, - CK ↑ - Phagocytic activity ↑ *Compared to Group A*, - URTI incidence ↑	Low Risk of Bias
Kowatari et al. 2001 [11]	22 male Judo athletes (18–21 years)	Intense Judo training + Group A: RWL within 20 days vs. Group B: non-RWL	- Body composition - Blood collection - Phagocytosis assays - Flow-cytometry	- BM ↓ - Body fat ↓ *Compared to Group B*, - Phagocytic Activity ↓	Low Risk of Bias
Hiraoka et al. 2019 [12]	30 male Judo athletes	Intense Judo training + Group A: 5% BW reduction within 3 weeks vs. Group B: 5% BW reduction within 3 weeks	- Body composition - Saliva sampling - URTI symptoms - Profile of mood states	- BM ↓ - BMI ↓ - Total body water ↑ *Compared to Group B*, - sIgA ↓ - URTI symptoms ↑ - Mood states ↓	Low Risk of Bias

* BMI: body mass index, ** HR: heart rate, *** BW: body weight, **** GH: growth hormone, ***** ELISA: enzyme linked immunosorbent assay, ^#^ ROS: reactive oxygen species, ^##^ RBC: red blood cells, ^###^ NO: nitric oxide, ^####^ RD: RBC deformability, ^#####^ RA: RBC aggregation, ^######^ DT: disaggregation threshold.

**Table 3:** Summary of cellular immune alterations induced by rapid weight loss in combat sports athletes.

Immune Cell Type	Observed Change	Potential Mechanisms	Clinical Implications	Ref.
Neutrophils	Count: Increase Function: Decrease	- Demargination: Cortisol and catecholamine surges mobilize neutrophils from bone marrow and vascular walls. - Functional impairment: Reduced oxidative burst capacity and phagocytic activity due to glycolytic ATP depletion and NADPH oxidase inhibition.	- False security: Elevated counts do not reflect functional competence. - Bacterial susceptibility: Diminished ability to clear extracellular bacteria, prolonging infection risk despite high cell numbers.	[4,10,11,24,25]
Lymphocytes (T-cells, B-cells)	Count: Decrease Proliferation: Decrease	- Apoptosis and redistribution: Glucocorticoids induce lymphocyte apoptosis and redistribution to lymphoid tissues. - Suppression: Downregulation of IL-2 and IFN-γ synthesis impairs clonal expansion upon antigen challenge.	- Adaptive immunity deficit: compromised long-term immunity and antibody response. - Viral risk: Increased vulnerability to viral pathogens and delayed recovery from viral infections.	[4,13,26,27]
NK cells	Cytotoxicity: Decrease	- Receptor downregulation: Cortisol suppresses activating receptors (e.g., NKG2D). - Energy deficit: lack of glucose/glutamine substrates impairs cytolytic granule exocytosis.	- Impaired surveillance: Reduced capacity to eliminate virally infected or stressed cells. - Early defense failure: Weakened first-line defense against intracellular pathogens.	[3,6,13]
Monocytes and Macrophages	Phenotype: Pro-inflammatory TLR Expression: Decrease	- M1 polarization: Stress hormones and metabolic endotoxemia drive monocytes toward inflammatory phenotypes (IL-6, TNF-α ↑). - TLR-4 suppression: Reduced expression of pathogen-recognition receptors (e.g., TLR-4) on monocytes.	- Systemic Inflammation: Promotion of a chronic, low-grade inflammatory state (sterile inflammation). - Delayed Repair: Impaired transition to M2 (anti-inflammatory) phenotype delays muscle tissue repair and resolution of inflammation.	[4,13,28,29]

**Table 4:** Immune parameters affected by rapid weight loss.

Immune Parameter	Consistent Findings	Effect Size/Threshold	Clinical Outcome	Ref.
Suppressed mucosal immunity (sIgA ↓)	sIgA secretion rate ↓ + parallel saliva flow ↓ = dehydration-mediated	>5% BW loss in 3–5 days	URTI symptoms ↑ (runny nose, sore throat, etc.)	[4,10,12]
Neutrophil dysfunction	- Count ↑ - Phagocytic activity ↓ - Oxidative burst ↓	All RWL protocols	Bacterial susceptibility ↑ (false security from elevated counts)	[4,10,11,13]
Decreased NK cell activity	- Cytotoxicity ↓ (cortisol-mediated receptor downregulation)	>5% BW loss	Viral surveillance failure ↑ (impaired early defense)	[3,13]
Systemic inflammation	IL-6, TNF-α ↑ (glycogen depletion → muscle IL-6 release)	Caloric restriction + dehydration	URTI risk ↑ (chronic dysregulation; delayed recovery)	[4,13,30]

## Data Availability

No new data were created or analyzed in this study. Data sharing is not applicable to this article.

## Supplementary Materials

The following supporting information can be downloaded at: https://www.mdpi.com/article/10.3390/ijms27010508/s1, Ref [109] is cited in the Supplementary Materials.

## Abbreviations

The following abbreviations are used in this manuscript:

RWL	rapid weight loss
URTI	upper respiratory tract infections
ROS	reactive oxygen species
NKG2D	natural killer group 2, member D
HPA	hypothalamic–pituitary–adrenal
sIgA	secretory immunoglobulin A
NK	natural killer
TNF	tumor necrosis factor
IL	interleukin
ACTH	adrenocorticotropic hormone
IFN	interferon
NADPH	nicotinamide adenine dinucleotide phosphate
ATP	adenosine triphosphate
TGF	transforming growth factors
APR	acute-phase response
APPs	acute-phase proteins
β2-AR	β2-adrenergic receptors
CR	caloric restriction
